# Differential Sensitivity of Prefrontal Cortex and Hippocampus to Alcohol-Induced Toxicity

**DOI:** 10.1371/journal.pone.0106945

**Published:** 2014-09-04

**Authors:** Anna-Kate Fowler, Jeremy Thompson, Lixia Chen, Marisela Dagda, Janet Dertien, Katina Sylvestre S. Dossou, Ruin Moaddel, Susan E. Bergeson, Inna I. Kruman

**Affiliations:** 1 Department of Pharmacology and Neuroscience, Texas Tech University Health Sciences Center, Lubbock, Texas, United States of America; 2 Laboratory of Clinical Investigation, NIA, National Institutes of Health, Baltimore, Maryland, United States of America; Radboud University, Netherlands

## Abstract

The prefrontal cortex (PFC) is a brain region responsible for executive functions including working memory, impulse control and decision making. The loss of these functions may ultimately lead to addiction. Using histological analysis combined with stereological technique, we demonstrated that the PFC is more vulnerable to chronic alcohol-induced oxidative stress and neuronal cell death than the hippocampus. This increased vulnerability is evidenced by elevated oxidative stress-induced DNA damage and enhanced expression of apoptotic markers in PFC neurons. We also found that one-carbon metabolism (OCM) impairment plays a significant role in alcohol toxicity to the PFC seen from the difference in the effects of acute and chronic alcohol exposure on DNA repair and from exaggeration of the damaging effects upon additional OCM impairment in mice deficient in a key OCM enzyme, methylenetetrahydrofolate reductase (MTHFR). Given that damage to the PFC leads to loss of executive function and addiction, our study may shed light on the mechanism of alcohol addiction.

## Introduction

Alcohol abuse is a serious health issue worldwide. Alcoholics experience a number of health problems including neurological and psychiatric disorders [1]. Postmortem studies of brain tissue in human alcoholics suggest that chronic heavy alcohol use transforms brain structure [2]. Brain-imaging studies support these observations [3], [4]. The use of alcohol and other drugs of abuse has been shown to lead to addiction, a chronic disease, frequently associated with neurological impairments such as cognitive dysfunction and impulsiveness [5]–[7]. At the cellular level, the effects of drugs of abuse including ethanol are often associated with oxidative stress resulting in oxidative damage to DNA and other biomolecules [8]. 8-oxo-7,8-dihydro-guanine (oxo-8dG) is an abundant product and a marker of oxidative DNA damage [9]. Excessive oxidative stress and subsequent DNA damage can be responsible for neuronal apoptosis and neuronal dysfunction associated with different neurological pathologies [10]–[12]. Ethanol-induced oxidative stress generates DNA damage which is reversible under conditions of acute ethanol exposure when cells are capable of repairing these DNA lesions [13]. In contrast to acute, chronic ethanol exposure is associated with reduced DNA repair [14], [15], and elevated blood homocysteine (Hcy) level, a marker of OCM dysfunction, is typical for alcoholics [14], [16], [17]. While OCM function is essential for DNA repair and the latter is critical for maintaining genomic stability [18], [19], genomic instability is central to carcinogenesis and is implicated in neurodegeneration [20], [21]. This can explain both carcinogenic and neurodegenerative effects of chronic alcohol abuse.

Both acute and chronic alcohol consumption affect the brain’s reward and executive control systems. According to neuroimaging studies, acute alcohol use selectively attenuates the anterior cingulate cortex (ACC) which has widespread anatomical connections with PFC, motor cortex, spinal cord, and limbic structures and thereby plays a versatile role in self-control [22], [23]. Chronic alcohol intake results in more persistent alterations in brain function and structure. Neurodegeneration and dementia are important features of chronic alcohol abuse [2], [24]. The frontal lobes of the cerebral cortex are particularly sensitive to alcohol-induced damage [2].

Increasing evidence suggests that repeated alcohol use functionally impairs the PFC which is responsible for highest-order cognitive function [6], [25]. It is unclear however, what causes this functional impairment. Damage to the PFC has been found to affect decision making abilities and lead to impulsive behavior [26], similar to behavioral features observed in individuals exposed to abuse substances including alcohol [6], [27]–[31]. Additionally, imaging studies demonstrated the link between structural PFC aberrations and addiction [32]–[34]. Thus, structural damage to the PFC may play a role in its functional impairment. In this context, understanding the mechanisms of alcohol-induced structural damage to PFC and identifying the threshold for this damage may be important for understanding the mechanisms of addiction as well. We suggest that the PFC neurons are selectively sensitive to alcohol toxicity, and alcohol-induced OCM impairment plays an important role in this neurotoxicity.

Here, we report that the PFC is more vulnerable to chronic alcohol-induced oxidative stress and neuronal death than hippocampus and that alcohol-induced OCM impairment plays an important role in structural PFC damage by causing genomic instability which leads to neuronal death. Neuronal death in the PFC, in turn, can determine the point when alcohol abuse causes addictive behavior.

## Materials and Methods

### Mice and Ethanol Exposure

This study was carried out in strict accordance with the recommendations in the Guide for the Care and Use of Laboratory Animals of the National Institutes of Health. The animal use protocol was approved by the Institutional Animal Care and Use Committee (IACUC) at the Texas Tech University Health Sciences Center, which oversees the use of laboratory animals for research purposes (IACUC approval number 08019). Ten to twelve-week-old C57BL/6 male mice (The Jackson Laboratory) ranged between 22–24 grams at the beginning of treatment were housed under standard conditions with a 12 h/12 h light-dark cycle and constant temperature (23±2°C). The animals were fed ad libitum a nutritionally adequate Lieber-DeCarli liquid diet containing 5% (v/v) ethanol or a control diet in which ethanol was substituted isocalorically with dextrin maltose (BioServ) for a period of 4 days (acute exposure), 3 weeks or 5 weeks (chronic exposure). Ethanol was introduced by increasing the content gradually by 1% (vol / vol) every day until the mice will be consuming diets containing 5% (vol /vol) ethanol, as described earlier [14]. Blood ethanol levels were determined using gas chromatography (GC), as described [35].

To specifically determine the role of OCM impairment in alcohol effects, we utilized methylenetetrahydrofolate reductase (MTHFR)- deficient mice with heterozygous (+/−) disruption of the gene (a breeding pair of these mice were kindly provided by Dr. Richard Finnell, UT at Austin). A shortage of this key OCM enzyme is expected to exaggerate alcohol effects on genomic stability and cell viability, as shown under conditions of OCM impairment induced by folate deficiency [36]. The mice were bred and PCR-genotyped as described [36]. Male *Mthfr*+/−10–12 week old mice were randomly assigned to one of the above treatment groups.

All mice were weighed daily and health monitored. Control and ethanol diets were made fresh each day and amount consumed each day was recorded. On the day of sacrifice, mice were weighed, fresh diet was given, and then four hours later diet was weighed off, and a 20 µl tail blood sample was taken for blood alcohol level analysis. The mice had no access to food or water for the next 6 hours, in order to accurately measure Hcy levels. Then the animals were sacrificed by cervical dislocation, trunk blood collected for Hcy analysis, and brains removed. Each brain was hemisected with either left or right side being fresh frozen for biochemical analyses, and the other side fixed in either Bouin’s or 4% paraformaldehyde for histochemical analyses.

For stereological studies, we used 2 treatment groups of animals (control and 3-week ethanol exposed), n = 8 for each group. For DNA repair, Hcy and immunofluorescence assays, we utilized WT mice, 4 treatment groups (control and ethanol exposed for 4 days or 3-and 5 weeks), n = 5 for each group and Mthfr+/− mice, 2 treatment groups (control and ethanol exposed for 3 weeks), n = 6 for each group.

### Homocysteine Analysis

The experiments were carried out as described earlier [14] using an HPLC system (Shimadzu Scientific Instruments). Hcy, acetonitrile, formic acid (Sigma), and dl- [^2^H_4_]Hcy (Cambridge Isotopes Laboratories) were utilized as standards. Hcy concentration was determined using [^2^H_4_]Hcy as the internal standard, where the concentration of the internal standard was set at 2000 ng/ml. Five µl of 20 µg/ml of [^2^H_4_]Hcy and 15 µl of water were added to 30 µl of plasma samples, and Hcy was separated using an Eclipse XDB-C18 guard and analytical columns, 4.6×12.5 mm and 150×4.6 mm inner diameter, 5 µm, Zorbax C18 Agilent, respectively. Mass spectrometry (MS/MS) assay was performed using a triple quadrupole mass spectrometer (API 4000 system) equipped with Turbo Ion Spray (Applied Biosystems). The data were analyzed using Analyst Version 1.4.2 (Applied Biosystems). The Turbo Ion Spray instrumental source settings for temperature were 200°C, 10, 60, and 60 p.s.i., and 5500 V, compound parameter settings for declustering, entrance, and collision cell exit potentials were 30, 10, and 12 V, respectively. The collision energy setting was 20 V. Hcy and [^2^H_4_]Hcy were characterized using the multiple reactions monitoring ion transitions 136.1–90 and 140.0–94, respectively. Hcy extraction was performed using 1 ml of solid phase extraction cartridges (Oasis MCX, Waters Corp.) preconditioned with 1 ml of methanol, followed by 1 ml of water. The plasma samples were washed with 1 ml of 1% formic acid and 1 ml of methanol, and the compounds were eluted with 1 ml of 5% ammonium hydroxide. The eluate was stream-dried under nitrogen at 60°C, reconstituted in 100 µl of methanol, and transferred to the autosampler vial for analysis.

### Histological Assays

Oxidative DNA damage and neuronal cell death were determined by histological assays as described [14]. Brains were collected, fixed in Bouin’s fixative, embedded in gelatin blocks (for stereology) or fixed by 4% paraformaldehyde and embedded in paraffin (for confocal microscopy). For stereology, gelatin blocks were serially sectioned in coronal planes at 25 µm by Neuroscience Associates, Inc. The sections were then subjected to antigen retrieval (ProHisto, 15 min). Endogenous peroxidase activity was blocked with 0.3% H_2_O_2_ in 100% methanol. One set of slides was stained using ‘In Situ Cell Death detection Kit’ (Roche; TUNEL, 11 684 795 910). The sections were permeabilized using 0.1% Triton X-100 in 0.1% sodium citrate. A positive control was performed by incubating with DNase I (Invitrogen; 18047-019) prior to labeling; a negative control was performed by incubating the slides with the TUNEL label in the absence of the enzyme. The second set of slides was incubated with antibody to oxo-8dG (1∶100; Abcam; ab64548). Both TUNEL and oxo-8dG labeling were combined with neuronal marker, MAP-2 (1∶500; Millipore; AB5622). Then sections were incubated with ImmPRESS reagent (Vector Laboratories; rabbit MP7401 and mouse MP7402) for 30 min and with DAB ImmPACT (Vector Laboratories; SK4105) in combination with 1.5% NiCl (10 min), and counterstained with Mayer’s hematoxylin (Electron Microscopy Services).

Stereological counting was carried out using the CASTGRID software (Olympus DK). An Olympus BX51 microscope with a motorized stage (x, y), attached microcator (z axis, Heidenhain), and color camera (Hitachi HV-C20) were used. Both the optical dissector and fractionator methods were employed to compare the two estimates of total cell number and relative efficiency. Cavalieri’s principle was used to determine the volumes of the PFC and the hippocampus (Vref). The density (Nv) of total labeled neurons, positive for oxo-8dG or TUNEL, was established using the optical dissector method: Nv = ΣQ−/Vdis•ΣPi, whereby ΣQ is the total number of cells counted; Vdis is the counting frame area multiplied by the height of the optical dissector, and ΣPi is the total number of stops within the reference volume counted. All counting was done with an oil immersion x 60 objective.

Coronal sections were also used for confocal microscopy analysis. Paraffin sections (10 µm) were deparafinized and fixed with 4% paraformaldehyde for 10 min. Then the sections were subjected to antigen retrieval (ProHisto, 15 min), treated with 0.3% H_2_O_2_ in 100% methanol to block endogenous peroxidase activity and stained for active caspase 3 (1∶500; R&D Systems AF835) and then for TUNEL using the In Situ Cell Death Detection kit (Roche Applied Science; 11 684 795 910). A positive control was performed by incubating with DNase I (Invitrogen; 18047-019) prior to labeling. Slides with the TUNEL label in the absence of the enzyme were utilized as a negative control. Primary antibody was then labeled with Alexa Fluor 647 (1∶2000; Life Technologies A21245). The sections were counterstained with a neuronal marker, NeuN conjugated with Alexa 555 (1∶100; Millipore MAB377A5). Cell nuclei were identified using Hoechst 33342 (Life Technologies; H1399).

Confocal imaging was performed using Olympus IX71 microscope equipped with a high temporal and spatial resolution EMCCD camera. All imaging settings were kept consistent across controls and ethanol-exposed groups.

### DNA Repair Assay

**Figure 1:**
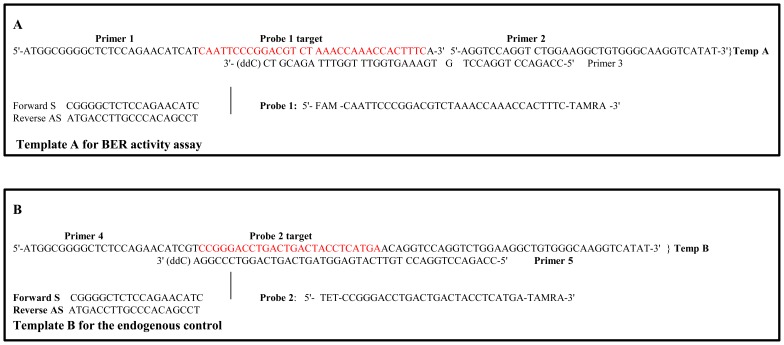
Templates for qPCR-based BER activity assay. Whole cell extracts were isolated from brain tissue and exposed to a template DNA containing a single nucleotide lesion (A) and control template (B). BER activity was calculated by comparing the ΔΔCt values (Ct is the number of cycles required for the fluorescent signal to cross the threshold) of the repaired and control templates.

DNA repair activity was analyzed using Real Time qPCR, as described previously [14], [37]. Whole cell extracts were obtained from brain tissue (PFC or hippocampus) using the kit from Active Motif, and total protein content was quantified by the Bradford assay. The lysates were incubated for 30 min at 32°C in a reaction mixture containing 2 pmol of DNA template A containing single nucleotide deletion, 2 pmol of endogenous control DNA template B (Fig. 1), 45 mm HEPES-KOH, 70 mm KCl, 7.4 mm MgCl2, 0.9 mm DTT, 0.4 mm EDTA, 2 mm ATP, 20 µl each of dATP, dTTP, cCTP, and dGTP, 40 mm phosphocreatine, 2.5 µg of creatine phosphokinase, 20 µg/ml BSA, 3.4% glycerol, 2 mm NAD+, and 4 µg of poly(dI/dC). The reaction was terminated by heating (72°C, 10 min). The probes and primers were designed as described [14] and synthesized by Gene Link. Two µl of a ×10,000 dilution of the reaction mixture was used as the template for qPCR with forward and reverse primers and a probe followed by qPCR monoplex reactions with annealing temperature of 60°C for 40 cycles. The quantity of repaired templates was calculated by comparing the ΔΔCt values (Ct is the number of cycles required for the fluorescent signal to cross the threshold) of the repaired and control templates.

### Statistical Analyses

Statistical analyses were performed with Microsoft Excel, and *p* values were obtained using ANOVA and Fisher’s post hoc test. All data are represented as mean ± standard error of the mean (SEM). A *p* value <0.05 was considered significant.

## Results

### Long-term chronic alcohol consumption induced a significant oxidative DNA damage in mouse prefrontal cortex, but not in hippocampus

**Figure 2:**
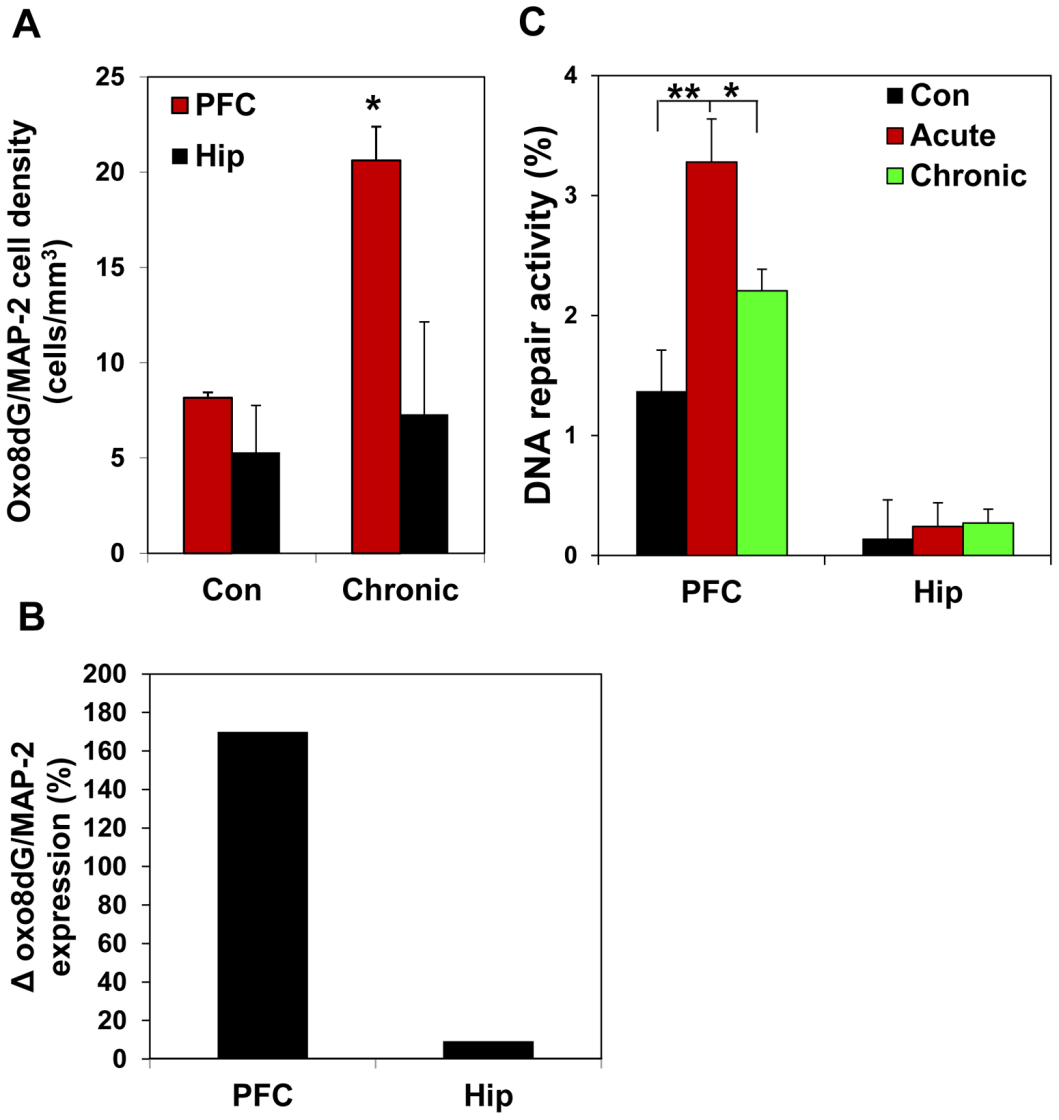
PFC is more vulnerable to ethanol-induced oxidative stress than hippocampus. (A) oxo-8dG expression in neurons (neuronal marker MAP-2) was quantified in PFC and hippocampus (Hip) by stereological counting. Neither the number of neurons nor volumes of the brain structures were affected. Values are mean ± SEM; *p<0.01. (B) Oxo-8dG expression in neurons normalized to corresponding controls (Δ). Note significantly higher density of oxo-8dG -labeled neurons and Δ in PFC, compared with hippocampus of ethanol-exposed mice. (C) DNA repair activity in response to oxidative DNA damage (oxo-8dG) assessed by qPCR in whole cell extracts obtained from PFC and hippocampus (Hip) of control mice and mice exposed to acute or chronic ethanol. Values are means ± SEM; *p<0.05, **p<0.01. Note the response to oxidative DNA damage by PFC lysate is significantly stronger than those in the hippocampus.

In the present study, we used a mouse model of chronic ethanol exposure to examine ethanol- induced changes in the PFC. We compared the effects of long-term chronic ethanol exposure of C57BL/6 mice on PFC and hippocampus. The C57BL/6 mouse is one of few inbred strains that allow consumption to reach biologically relevant blood alcohol levels [35]. The consumption of control liquid Lieber-DeCarli diet did not significantly differ from the consumption of those containing 5% ethanol. Blood ethanol levels in all groups of animals exposed to ethanol were 53.4±6.4 mM (254.4±33.2 mg%). Using stereological counting, we determined the density of neurons positive for oxo-8dG, a marker of oxidative DNA damage. For identification of neurons, we utilized neuronal marker MAP-2. Oxo-8dG- positive neurons were quantified throughout the entire PFC and hippocampus, as we described before [14]. Three-week ethanol exposure significantly increased the number of oxo-8dG (1886264.3±362181.1 in ethanol group versus 798319.3±18449.2 in control group; p = 0.033), and the density of such neurons in PFC, but not in hippocampus, where ethanol affected neither the number of oxo-8dG- positive neurons (606996.9±235816.4 in ethanol group versus 543881.5±55076.1 in control group; p = 0.388), nor their density (Fig. 2A). The difference in alcohol effects on PFC and hipppocampus is seen even better on Fig. 2B, where the density of oxo-8dG expression in neurons is normalized to corresponding controls. We also analyzed a capacity of the PFC and hippocampus tissue lysates to repair alcohol-produced oxidative DNA damage using qPCR (Fig. 1). To accomplish this goal, we included an additional treatment group, mice exposed to ethanol acutely (4 days). Previously, we demonstrated that acute ethanol exposure does not activate the expression of apoptotic markers but notably activates DNA repair in the cerebral cortex [14] which is a typical response to DNA damage [38]. This is not surprising, given that alcohol metabolites are genotoxic [13], [18]. In healthy cells, the rate of DNA repair is equal to the rate of DNA damage which allows to maintaining genomic stability and viability [39]. We therefore utilized this group of treatment to compare responses of PFC and hippocampus to acute alcohol-produced oxidative DNA damage. A higher DNA repair response in the PFC, compared with hippocampus is a measure of increased oxidative DNA damage and enhanced oxidative stress in this region. The higher response to oxidative DNA damage (and oxidative stress) in PFC, compared with those in the hippocampus (Fig. 2C) suggests that PFC is more affected by ethanol-produced oxidative DNA damage than hippocampus and is more vulnerable to alcohol-produced oxidative stress.

### Long-term chronic alcohol consumption induced a significant increase in the density of TUNEL-labeled neurons in mouse PFC but not in hippocampus

**Figure 3:**
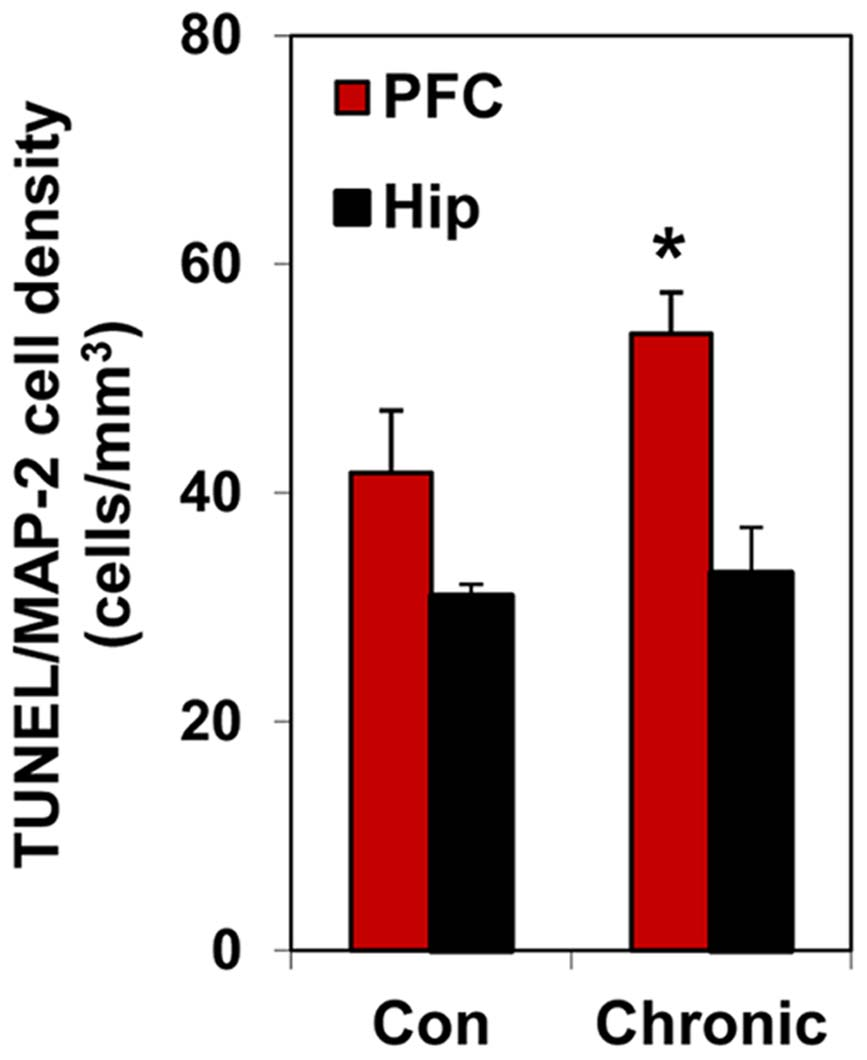
PFC is more vulnerable to ethanol-induced neuronal apoptosis than hippocampus. Brain sections double-labeled with TUNEL and MAP-2 in the PFC and hippocampus (Hip) and quantified by stereological counting; Values are means ± SEM; *p<0.01. Neither the number of MAP-2-positive cells (neurons) nor volumes of the brain structures were affected by chronic 3-week ethanol exposure. Note significantly higher density of TUNEL- positive neurons in PFC than in hippocampus of ethanol-exposed mice.

Since PFC neurons revealed a significant increase in markers of oxidative stress following chronic alcohol exposure, and oxidative stress is directly linked to neuronal apoptosis [10]–[12], we tested whether the selective vulnerability of PFC neurons to alcohol-induced oxidative stress would result in its selective vulnerability to neuronal death. Using stereological counting of neurons (MAP-2 labeling) expressing apoptotic marker, terminal transferase-mediated dUTP-biotin nick end-labeling (TUNEL), we thereby quantified the density of apoptotic neurons in the PFC and the hippocampus. Our results demonstrate that a three-week chronic ethanol exposure caused a significant increase in the number of TUNEL positive neurons (4157454.6±467529.7 in ethanol group versus 3211247.0±392185.1 in control group; p = 0.048) and their density in the PFC. Conversely, the number of TUNEL-positive neurons (456937.9±47408.5 in ethanol group versus 426548.2±97584.6 in control group) or their density in the hippocampus was not affected over this same period of alcohol treatment (Fig. 3). In contrast to the density of TUNEL-positive neurons, the total neuronal density in the PFC was not influenced by chronic alcohol (601644.1±58266.1 in ethanol group versus 590644.0±19937.6 in control group; p = 0.424). Thus, three-week alcohol exposure resulted in neuronal apoptosis, but not neuronal loss.

**Figure 4:**
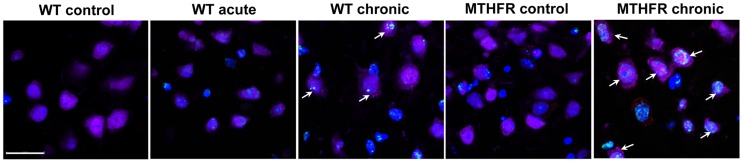
OCM impairment is involved in ethanol-induced oxidative DNA damage and neuronal apoptosis effects in the PFC. The brain sections (PFC) of WT and *Mthfr+/−* mice exposed for 3 weeks or 4 days (acute) to the Lieber-DeCarli liquid diet with- or without ethanol (5%) were triple-labeled with NeuN (purple), TUNEL (green) and cleaved caspase-3 (red). Hoechst 33342 (blue) was used to identify all cell nuclei. Fluorescence was visualized by confocal microscopy. Scale bar = 20 µm. Note increased number of TUNEL/caspase-3-positive neurons in PFC of *Mthfr+/−* mice chronically exposed to ethanol, compared with corresponding WT mice (arrows). Also, note a higher density of TUNEL/caspase-3-positive neurons in PFC of chronically, compared with acutely exposed to ethanol WT mice (arrows).

For more specific identification of apoptosis, we utilized double-labeling of neurons with TUNEL and another apoptotic marker, active caspase-3, since neither of these markers is strictly specific for apoptosis. TUNEL may also label cells undergoing non-apoptotic cell death and theoretically can mark DNA repair and gene transcription [40], [41], although the sensitivity of TUNEL labeling is very limited for such assessments [42], [43]. Active (cleaved) caspase-3, besides its critical involvement in apoptosis, has been found to play roles in cell cycle, differentiation and proliferation [44], [45]. However, the co-incidence of these two independent apoptotic markers is more reliable for identification of apoptosis. Concomitant expression of TUNEL and active caspase-3 in neurons seen in microphotographs demonstrates that in contrast to acute, chronic ethanol consumption induces neuronal caspase-dependent apoptosis in the PFC (Fig. 4).

### OCM impairment and neurotoxic effect of ethanol

**Figure 5:**
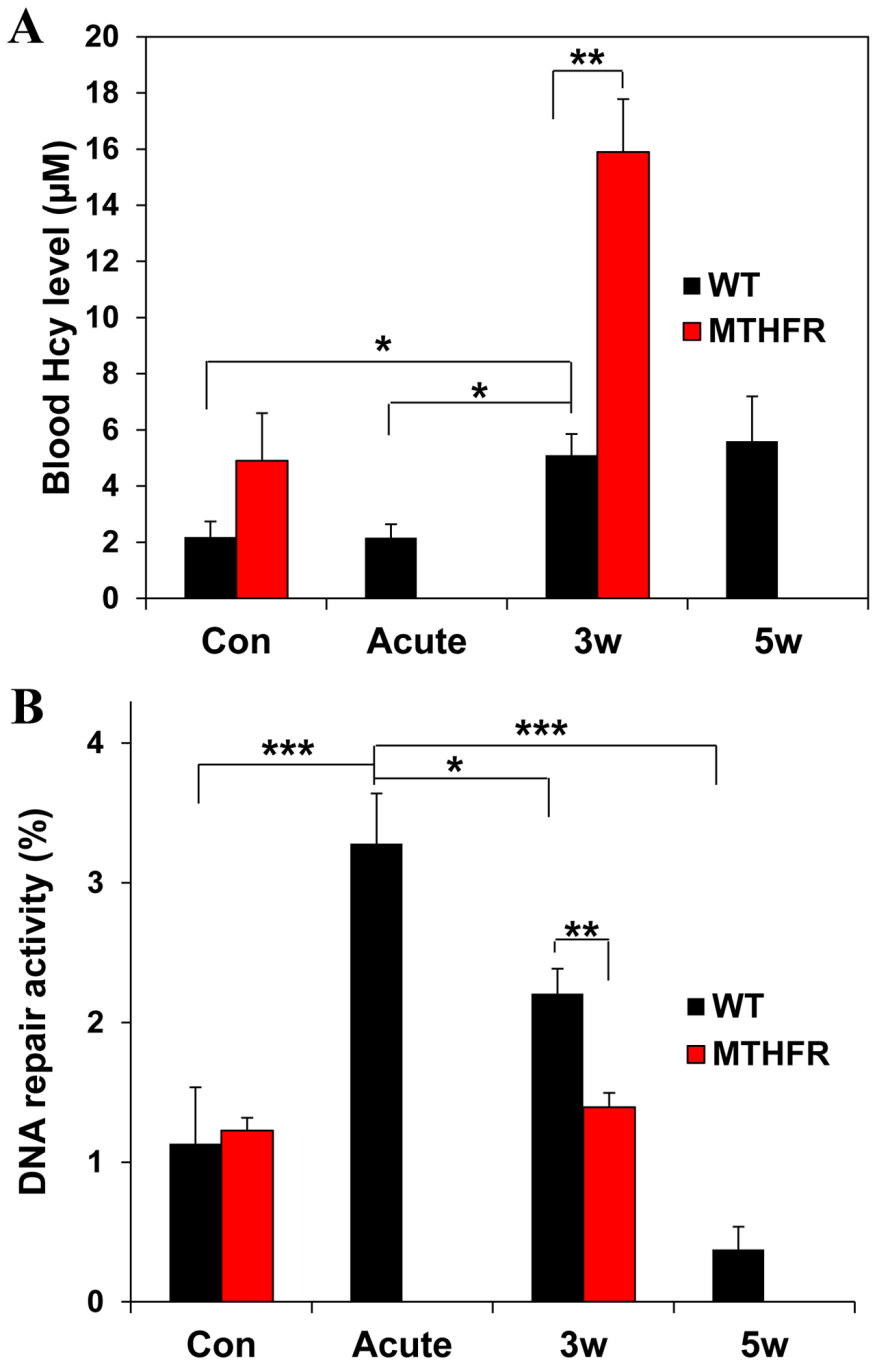
OCM impairment is involved in ethanol impact on DNA repair in the PFC. (A) Blood Hcy levels in WT and *Mthfr+/−* mice following acute or chronic 3- week (3 w) or 5-week (5 w) ethanol exposure. Values are means ± SEM; *p<0.01; **p<0.001. Note chronic alcohol-induced increase in blood Hcy levels, compared with the acute exposure and the heightening this increase by MTHFR deficiency. (B) DNA repair activity in the PFC of WT and *Mthfr+/−* mice exposed to acute and chronic ethanol. Values are means ± SEM; *p<0.01; **p<0.005; ***p<0.002. Note a decrease in DNA repair activity in the PFC following 3-week (3 w) and even more so following 5 week (5 w) exposure, compared with acute alcohol exposure and a significant exaggeration of this decrease by MTHFR deficiency.

To determine the involvement of OCM impairment in alcohol effects on PFC, we examined the impact of additional OCM impairment caused by deficiency in the key OCM enzyme MTHFR in *Mthfr+/−* mice on alcohol-induced damage to the PFC. Our results show that MTHFR deficiency increased the number of TUNEL/active caspase-3-positive neurons in the PFC of these mice, compared with WT mice, suggesting that additional OCM impairment in Mthfr+/− mice exaggerated damaging effect of chronic alcohol on PFC neurons (Fig. 4). Three- and five-week alcohol exposure led to a significant increase in blood Hcy levels in WT mice. MTHFR deficiency in *Mthfr+/−* mice enhanced effect of chronic alcohol exposure, while under control conditions, MTHFR deficiency did not significantly affected Hcy levels. Acute alcohol exposure did not affect Hcy levels as well (Fig. 5A). These data suggest a link between OCM function (elevated Hcy levels are a marker of OCM impairment) and damaging effect of alcohol on PFC neurons. OCM is responsible for synthesis of DNA precursors and is the major source of methyl groups for DNA and histone methylation, which is an important epigenetic determinant in gene expression. OCM impairment leads to DNA repair dysfunction and aberrant DNA methylation, both of which impact genomic integrity [46], [47]. We therefore examined the impact of OCM status on DNA repair capacity in the PFC. Acute alcohol exposure activated DNA repair, while DNA repair capacity in chronically exposed mice was reduced, compared with those in mice with acute treatment (Fig. 5B). A longer (5 weeks) exposure exaggerated this alcohol effect on DNA repair. Additional OCM impairment induced by MTHFR deficiency in Mthfr+/− mice sensitized them to ethanol-induced DNA repair dysfunction and also compromised DNA repair, as compared with alcohol alone for the same treatment period (Fig. 5B). Meanwhile, under control conditions, DNA repair was not affected by MTHFR deficiency. Together, these data reinforce the significance of OCM impairment in alcohol-induced damage to the PFC.

## Discussion

The main objective of our study was to gain a better understanding of the potential contribution of selective PFC damage and OCM dysfunction to its alcohol-induced neurological impairments. Here, we show that the PFC is more susceptible to alcohol-induced oxidative stress and neuronal apoptosis than the hippocampus, as seen from significantly increased densities of apoptotic neurons and neurons containing oxidative DNA damage in the PFC following long-term chronic alcohol exposure. This suggests that differential PFC damage can lead to deficits in its function. Our data suggest a link between ethanol interference with OCM and neuronal cell death in the PFC. Chronic, in contrast to acute ethanol exposure, disturbed OCM function, as evidenced by elevated blood Hcy levels and reduced DNA repair capacity, compared with acute treatment. Reduced DNA repair capacity is coupled with genomic instability [46] which has an important impact on neuron viability [14]. Our results obtained with *Mthfr+/−* mice support the involvement of OCM disturbance in alcohol-induced PFC damage, demonstrating that additional OCM impairment generated by MTHFR deficiency exaggerated the effects of ethanol on DNA repair and neuronal cell death in the PFC. These results emphasize the significance of OCM impairment in alcohol-induced damage to the PFC. Given that homozygous *MTHFR 677C>T* mutation, common in human population is associated with reduction in MTHFR activity similar to those in *Mthfr +/−* mice [36], *MTHFR 677C>T* polymorphism may be considered a risk factor for alcohol-induced structural PFC damage.

Damage to the ventromedial prefrontal cortex (vmPFC) has been shown to result in poor decision making abilities and impulsive behavior [26]. Similar behavioral features have been observed in individuals exposed to abuse substances including alcohol [6], [27]–[31]. Thus, the PFC, which is responsible for executive functions and inhibition of impulsive responses, is functionally impaired in substance abusers including alcoholics. This functional PFC impairment may result from drug-induced neurotoxicity and associated structural abnormalities, as it has been shown in imaging studies [34], [48]. The neurotoxic effects of drugs of abuse may be enhanced by other factors such as stress, which has been well known to increase vulnerability to addiction [49], [50]. PFC is a primary target for both stress and drugs of abuse [26], [51]. Even mild acute stress can cause a significant loss of prefrontal cognitive abilities. A chronic stress, specifically, social isolation stress, has been shown to induce oxidative stress and proapoptotic signaling in the PFC, while the hippocampus was significantly less affected [52], [53]. These stress effects are mediated by glucocorticoids, which cause an increased vulnerability to excitotoxicity [54]. Thus, the enhancement of drug abuse effects under stress conditions may be explained by additive damaging effects of drugs and stress on PFC, leading to accelerated structural PFC damage and thereby increasing vulnerability to addiction. The important role of structural PFC damage in addiction has also been shown by Pelloux and colleagues [34]. They demonstrated that selective damage to distinct PFC subregions accelerated transition to addictive behavior in rats after a short period of cocaine taking at a time when it would not usually be revealed.

While the structural damage to the PFC underlies the decision-making impairment observed in alcoholic patients, our data demonstrate that chronic alcohol exposure induces selective PFC damage, and OCM impairment plays a critical role in this damage. In contrast to chronic, acute alcohol consumption did not disturb OCM and did not induce neuronal cell death in the PFC. It is also known that in contrast to chronic, acute alcohol use is rarely associated with addiction [7], [55]–[57]. This supports a link between selective PFC damage and addiction and suggests a potential role of OCM impairment in controlling the point of transition to apoptotic cell death as a mechanism of developing addictive behavior. Since the structural damage to the PFC could be one of the critical mechanisms underlying the transition to addiction, our results suggest the mechanism involved in structural PFC damage which may also be involved in the development of addictive behavior. Future experiments aiming to investigate the potential direct link between neuronal damage in the PFC and addictive behavior will facilitate a better understanding of the mechanism of addiction.
